# Renoprotective Effect of *Laminaria japonica* Polysaccharide in Adenine-Induced Chronic Renal Failure

**DOI:** 10.3390/molecules24081491

**Published:** 2019-04-16

**Authors:** Miao Long, Qiang-Ming Li, Qing Fang, Li-Hua Pan, Xue-Qiang Zha, Jian-Ping Luo

**Affiliations:** 1 Engineering Research Center of Bio-process of Ministry of Education of China, Hefei University of Technology, Hefei 230009, China; longmiao0218@163.com (M.L.); liqm_2010@163.com (Q.-M.L.); inuyawelong@sina.com (Q.F.); panlihua@hfut.edu.cn (L.-H.P.); 2 School of Food and Biological Engineering, Hefei University of Technology, Hefei 230009, China; 3 Key Laboratory of Metabolism and Regulation for Major Disease of Anhui Higher Education Institutes, Hefei University of Technology, Hefei 230009, China

**Keywords:** *Laminaria japonica*, polysaccharide, chronic renal failure

## Abstract

Chronic renal failure (CRF) is a major public health problem worldwide. In this work, we investigated the effects of a purified *Laminaria japonica* polysaccharide (LJP61A) on renal function using an adenine-induced CRF mice model. Results exhibited that adenine treatment caused serious renal pathological damages and elevation of serum creatinine and blood urea nitrogen of mice. However, these changes could be significantly reversed by the administration of LJP61A in a dose-dependent manner. Additionally, LJP61A could dramatically reduce weight loss, improve the urine biochemical index, and regulate the electrolyte disturbance of CRF mice. These results suggest that the renal function of adenine-induced CRF mice can be improved by LJP61A, which might be developed into a potential therapeutic agent for CRF patients.

## 1. Introduction

Chronic renal failure (CRF) is a type of kidney disease characterized by a slow and progressive decline in renal function leading to irreversible kidney sclerosis and nephron loss [1]. In recent years, the morbidity and mortality of CRF is rising markedly, which has brought a heavy financial burden to our society [2,3]. In China, there are 119.5 million CRF patients in 2012, with an overall prevalence of 10.8% [4]. The main therapeutic modalities for end-stage CRF are hemodialysis and kidney transplantation [5]. However, patients treated with hemodialysis are reported to have a higher overall risk of developing cancer when compared to the general population [6]. Additionally, although there have been advances in kidney transplantation, limited available sources of kidneys restrict its application [5]. Therefore, it is imperative to develop new and effective agents from herbs or edible medicinal materials to treat or alleviate CRF.

**Figure 1:**
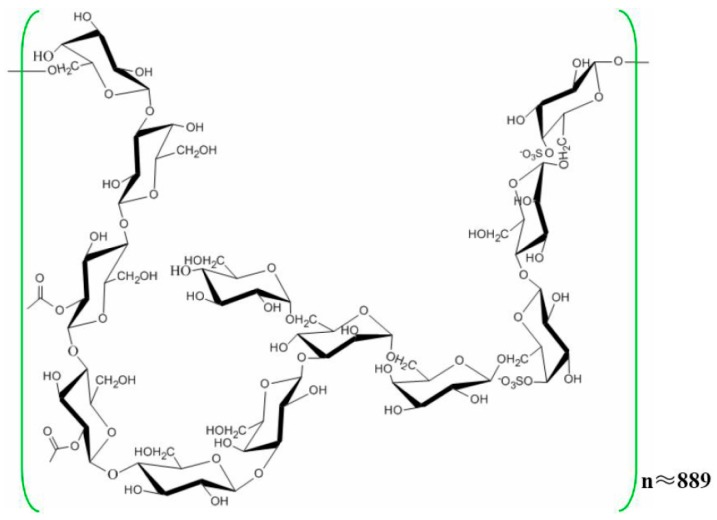
The chemical structure of LJP61A.

*Laminaria japonica*, a common economic edible-medicinal marine vegetable, has long been used as an important therapeutic agent for phlegm elimination and detumescence in China [7,8]. In recent years, the functional components of *L. japonica* have been widely reported by chemists and pharmacologists [9,10]. Among these components, polysaccharides have been considered to be the main active components, and have a wide range of biological activity, such as anti-tumour, anti-virus, anti-oxidant and anti-radiation effects [11,12]. In our previous works, a purified *L. japonica* polysaccharide (LJP61A) was isolated and characterized as a repeating unit consisting of →3,6)-α-d-Manp-(1 →, →4)-α-d-Manp-(1 →, →4)-2-*O*-acetyl-β-d-Glcp-(1 →, →4)-β-d-Glcp-(1 →, →6)-4-*O*-SO3-β-d-Galp-(1 →, →6)-β-d-Galp-(1 →, →3)-β-d-Galp-(1 →, and a terminal residue of α-d-Glcp-(1→ (Figure 1). [13]. LJP61A has been proven to suppress atherosclerosis via the regulation of cellular lipid metabolism, inhibition of cellular inflammation and alleviation of insulin resistance [13,14]. Additionally, we demonstrated that LJP61A could ameliorate vascular calcification via preventing osteoblastic differentiation of vascular smooth muscle cells [15]. Because atherosclerosis and vascular calcification are closely related to the development of CRF [16,17], we speculated that LJP61A might have the potential for treating or alleviating CRF. Therefore, the protective effects of LJP61A on renal function were investigated in the present work using an adenine-induced CRF mice model.

## 2. Results

### 2.1. LJP61A Regulates Body Weight, Water and Food Intake of CRF Mice

**Figure 2:**
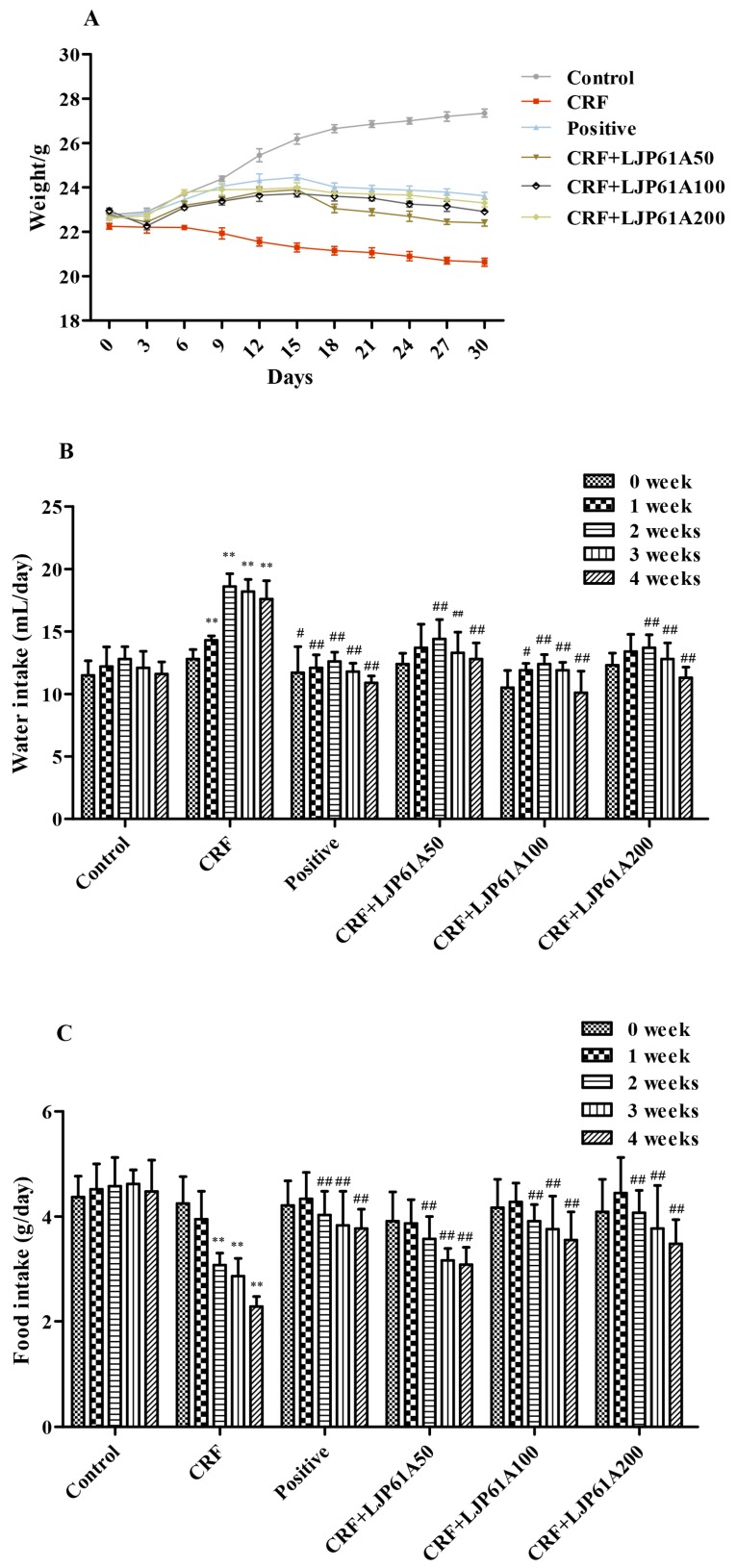
The effects of LJP61A on the body weight (**A**), water (**B**) and food (**C**) intake of adenine-induced CRF (chronic renal failure) mice. ** *p*< 0.01 (vs. Control group); # *p*< 0.05, ## *p*< 0.01 (vs. CRF group).

As shown in Figure 2A, the body weights of CRF mice were significantly reduced by adenine when compared with those of the control group. However, the weight loss of CRF mice could be attenuated by LJP61A. In addition, it was found that adenine treatment remarkably increased the water intake of CRF mice, and reduced the food intake (Figure 2B,C). However, these alterations of CRF mice were significantly reversed by LJP61A. These results indicated that the physiological state of adenine-induced CRF mice could be enhanced by LJP61A. Simultaneously, the effect of using a cinacalcet positive group was also very significant.

### 2.2. LJP61A Ameliorates Kidney Injury of CRF Mice

**Figure 3:**
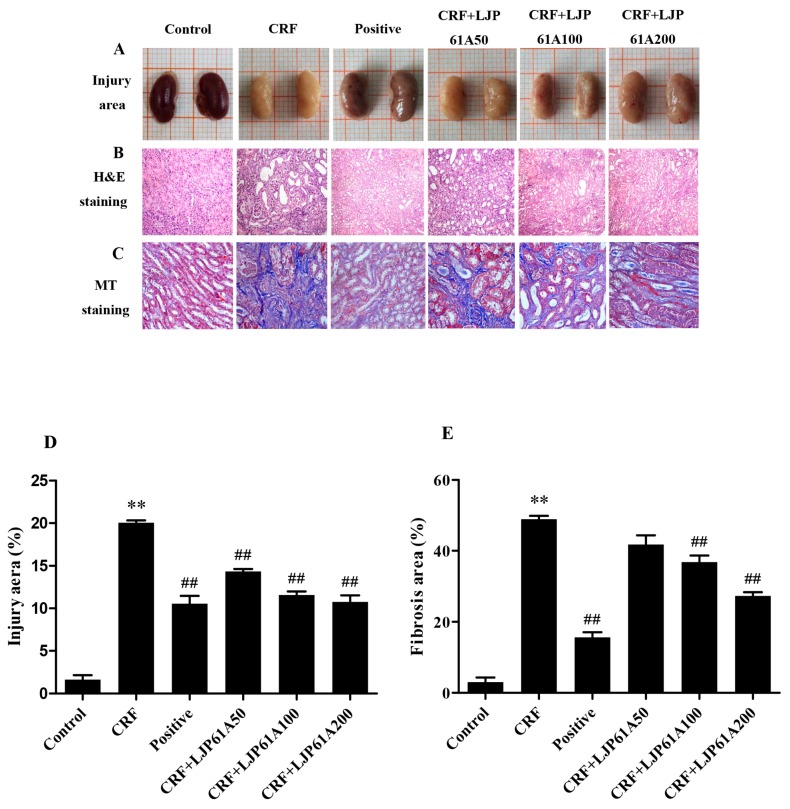
LJP61A ameliorates kidney injury in adenine-induced CRF mice. (**A**) Renal morphology; 10mm/grid (**B**) H&E staining; (**C**) MT staining; (**D**) injury area; (**E**) fibrosis aera. ** *p*< 0.01 (vs. Control group); ## *p* < 0.01 (vs. CRF group).

As shown in Figure 3A, the kidneys of mice in the normal control group were reddish brown and glossy, while those of mice in CRF group were gray white and uneven. However, these pathological characteristics were ameliorated by LJP61A treatment, indicating LJP61A could mitigate the kidney injury of adenine-induced CRF mice. Hematoxylin-Eosin (H&E) and masson trichrome (MT) staining are the common methods to show the pathological changes of renal tissue and the extent of fibrotic tissue proliferation [18]. Figure 3B shows H&E stained sections of different kidneys. The purple area represents an accumulation of inflammatory cells. The results showed a dramatic increase of inflammatory cells in the kidneys of mice in the model group (Figure 3D). Compared to the model group, inflammatory cell accumulation was inhibited by LJP61A treatment in a dose-dependent manner (Figure 3D). Figure 3C shows the Masson’s trichrome stained sections of different kidneys. A dramatic increase in collagen deposition (blue) was observed in the kidneys of mice in the model group. However, this deposition was attenuated by LJP61A (Figure 3E). When LJP61A dosage reached 200 mg/kg/day, the areas of injury and fibrosis were suppressed by 44.7% and 43.8% compared to those of the CRF group, respectively. For the positive control group, cinacalcet also showed inhibitory effects on adenine-induced kidney injury.

### 2.3. LJP61A Regulates the Blood Biochemical Index of CRF Mice

**Figure 4:**
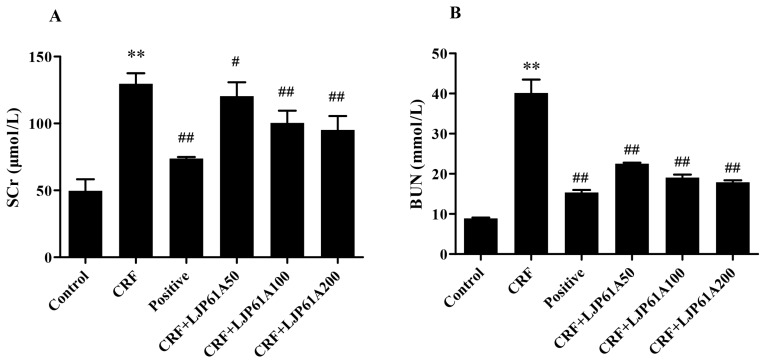
The effects of LJP61A on the SCr (**A**) and BUN (**B**) levels of adenine-induced CRF mice. ** *p* < 0.01 (vs. Control group); # *p* < 0.05, ## *p* < 0.01 (vs. CRF group).

Serum creatinine (SCr) and Blood urea nitrogen (BUN), two main end products of protein metabolism, are mainly excreted by glomerular filtration. They are the most important indexes of renal function [19,20]. As shown in Figure 4A,B, the SCr and BUN levels of mice in the CRF group were significantly elevated by adenine when compared with those of the control group. However, these enhancements were remarkably inhibited by LJP61A in a dose-dependent manner. When the dosage reached 200 mg/kg/day, the levels of SCr and BUN decreased to 73.41% and 44.62% of the CRF group, respectively. These results confirmed that LJP61A could improve the kidney function of adenine-induced CRF mice. At the same time, cinacalcet also decreased the levels of SCr and BUN compared to the model group.

### 2.4. LJP61A Regulates the Urine Biochemical Index of CRF Mice

**Figure 5:**
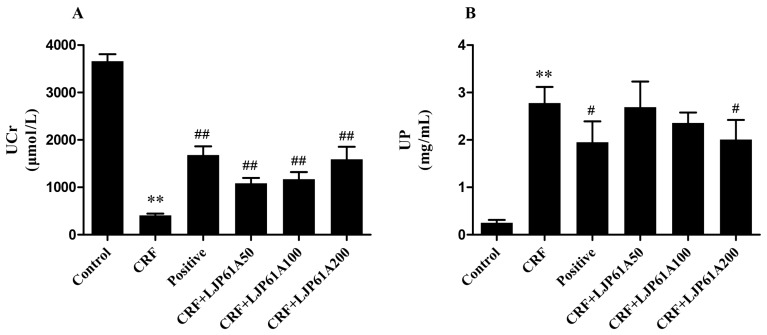
The effects of LJP61A on the UCr (**A**) and UP (**B**) levels of adenine-induced CRF mice. ** *p* < 0.01 (vs. Control group); # *p* < 0.05, ## *p* < 0.01 (vs. CRF group).

Besides SCr and BUN, urine creatinine (UCr) and urine protein (UP) are also considered to be key indexes of kidney function [21]. As shown in Figure 5, the UCr level of mice in the CRF group was significantly reduced by adenine when compared with that of the control group, while UP was increased. However, these alterations of CRF mice were significantly reversed by LJP61A, which further confirmed LJP61A could improve the kidney function of adenine-induced CRF mice. In the meantime, cinacalcet also significantly changed the levels of UCr and UP compared to the model group.

### 2.5. LJP61A Regulates the Electrolyte Disturbance of CRF Mice

Electrolyte disturbance is an important clinical manifestation of CRF [1]. Thus, the effects of LJP61A on the electrolyte disturbance of CRF mice were investigated. As shown in Table 1, compared with the control group, the CRF group showed a significant increase in serum levels of chlorine, potassium, magnesium, sodium and phosphorus. Meanwhile the urine levels of calcium, phosphorus and magnesium and the serum level of calcium significantly decreased. However, these alterations in the CRF mice were reversed by LJP61A, except for the serum levels of chlorine and sodium. These results indicate that LJP61A could improve the electrolyte disturbance of adenine-induced CRF mice. With respect to the positive control group, cinacalcet also showed an ability to suppress electrolyte disturbance.

## 3. Discussion

The kidney is an organ that performs a number of essential functions in the body: the clearance of endogenous waste products, the control of volume status, the maintenance of electrolyte and acid–base balance, and endocrine function [22]. When the kidney is damaged, the metabolism is disturbed, leading to electrolyte and acid-base imbalance [23]. In clinical trials, cinacalcet is a common therapeutic agent for CRF. Cinacalcet is a second generation calcimimetic agent used to sensitize calcium receptors on the parathyroid glands. Decreased levels of parathyroid hormone are associated with regression of left ventricular hypertrophy, thereby reducing cardiovascular calcification and chronic renal failure mortality [24]. Although this medication has a very wide applicability, cost is a major drawback that limits its utilization [25]. In the present study, we found that the fibrosis proliferation in the renal interstitium and brown purine crystal deposition in renal tubules and interstitium were observed in the adenine-induced CRF mice. These results were consistent with the pathological signs of CRF, indicating the CRF mice model induced by adenine was successful [26]. In comparison with the mice in the model group, LJP61A at 200 mg/kg/day alleviated digestive and absorptive dysfunction, the reduction of food intake and the increase of water intake caused by 0.25% adenine fed.

It has been reported that CRF can cause oxidative stress, inflammation, oxidation of lipoproteins and accelerated atherosclerosis [27,28]. Moreover, foam cells and atherosclerosis were also recognized to be related to the pathogenesis of kidney disease. Foam cells and macrophages are two key participants in atherosclerosis [29]. In our previous work, we also found LJP61A can prevent the conversion of macrophages into foam cells via modulating the expression of genes involved in regulation of the balance between cholesterol uptake and efflux, resulting in the suppression of atherosclerosis development [13]. For this action, the PPARγ pathway plays an important role. Furthermore, LJP61A was observed to attenuate ox-LDL-induced cell inflammation via mTOR, MAPKs, and NFκB signaling pathways [13]. In the present study, it can be seen from H&E stained sections that LJP61A can alleviate tubular damage in adenine-induced CRF mice by reducing the accumulation of inflammatory cells in the kidney. Therefore, we hypothesized that LJP61A may attenuate tubular damage in adenine-induced CRF mice by inhibiting the conversion of macrophages into foam cells to attenuate the accumulation of inflammatory cells in the kidney.

In the routine examination of renal function, the levels of blood urea nitrogen and serum creatinin are common clinical indexes to evaluate the glomerular filtration function, the reabsorption ability of renal tubules and the staging of CFR [20]. In many reports, acute kidney injury (AKI) causes a severe condition associated with high probabilities of developing progressive chronic kidney disease or end-stage renal disease, thus leading to high mortality rates. Currently, AKI is defined as an absolute increase in Scr levels of at least 0.3 mg/dL or a relative Scr increase of more than or equal to 50% within 48 h [22]. The decrease of BUN and Scr levels indicated that the renal function improved and the glomerular filtration capacity was enhanced. In the present study, LJP61A intervention can significantly reduce the content of urea BUN and Scr compared with those mice in the model group. We propose that LJP61A accelerated the metabolism of toxins, leading to the alleviation of azotemia, enhanced glomerular filtration power and suppression of renal function damage. These results suggest that LJP61A might delay the development of AKI to CRF.

Nagano et al. [30] reported that the glomerular filtration rate and renal tubule reabsorption capacity gradually decreased in CRF patients, resulting in a significant increase in urine protein and imbalance of electrolyte metabolism, especially increased blood phosphorus. It is a fact that persistent hyperphosphatemia is the leading cause of death in CRF patients as it causes the calcification of soft tissue and blood vessels, leading to heart and lung system diseases [31]. In our previous works, in the adenine-induced chronic renal failure (CRF) mice vascular calcification (VC) model and the β-glycerophosphate (β-GP)-induced vascular smooth muscle cells (VSMC) calcification model, LJP61A was found to significantly inhibit VC phenotypes. We also found that LJP61A remarkably up-regulated the mRNA levels of VSMC related markers and down-regulated the mRNA levels of the sodium-dependent phosphate cotransporter Pit-1. In addition, LJP61A can significantly decrease the protein levels of core-binding factor-1, osteocalcin, bone morphogenetic protein 2, and receptor activator for nuclear factor-κB ligand, and it can increase the protein levels of osteoprotegerin and matrix gla protein [15]. In the present study, there was a significant improvement of the electrolyte metabolic disorder in the urine and an increase of UP by the administration of LJP61A. These results suggest that LJP61A alleviated the impairment of renal tubular function induced by adenine, and that it relieved the symptoms of proteinuria induced by dysfunction of proximal tubule reabsorption. Moreover, LJP61A could regulate the serum levels of calcium, phosphorus and magnesium, resulting in stabilizing the serum electrolyte level and preventing further deterioration of the disease. Therefore, we hypothesized that LJP61A may reduce the adenine-induced increase in blood phosphorus by significantly down-regulating the mRNA levels of the sodium-dependent phosphate cotransporter Pit-1 to regulate electrolyte disturbance and improve renal function.

## 4. Materials and Methods

### 4.1. Chemicals

LJP61A was extracted and purified as described previously [14]. Adenine was purchased from Biosharp (Hefei, China). Cinacalcet tablets were purchased from Kyowa Hakko Kirin Co., Ltd. (Tokyo, Japan). All other reagents were analytical grade and obtained locally.

### 4.2. Animals

Eight-week-old male C57/BL6 mice (20 ± 2 g) were purchased from the Laboratory Animal Center of Anhui Medical University. The mice were maintained under specific pathogen-free conditions with a 12:12 h light–dark cycle at 25 ± 2 °C and 40% relative humidity. All animal handling procedures were performed strictly in accordance with the P.R. China Legislation on the Use and Care of Laboratory Animals.

### 4.3. Experimental Procedure

After an acclimatization period of one week, mice were randomly divided into six groups (20 mice per group), including a control group, CRF group, positive group, and LJP61A groups (CRF+LJP61A50, CRF+LJP61A100 and CRF+LJP61A200). The control group was fed a normal diet, while the others groups were fed the diet supplemented with adenine at the dose of 0.2% (*w/w*). The positive group was administered a cinacalcet tablet at the dosage of 150 mg/kg/day. The control and CRF groups were administered with the same volume of physiological saline. The LJP61A groups were orally administered LJP61A at 50, 100 and 200 mg/kg/day, respectively. Meanwhile, all mice were weighed weekly during the experimental period. After five weeks feeding, all mice were euthanized with CO_2_. Serum and kidneys were collected for the analysis of blood biochemistry and pathology.

### 4.4. Water and Food Intake

Five mice were randomly selected to be placed in the metabolic cage every week. Pellet feed (15 g) was put into the trough and 50 mL water was put into the water hole. Meanwhile, the urine cup and the fecal cup were placed in the cage. After 24 h, the residual feed and water were weighed to analyze the food and water intake.

### 4.5. Kidney Pathological Examination

The kidneys were fixed in 4% paraformaldehyde, dehydrated in increasing concentrations of ethanol, cleared with xylene and embedded in paraffin. From the paraffin blocks, five micrometer sections were prepared and stained with hematoxylin-eosin (H&E) and masson trichrome (MT) staining in order to observe the areas of injury (purple) in renal tissue and degrees of fibrosis tissue hyperplasia (blue) [32,33].

### 4.6. Serum Parameters

The serum creatinine (SCr), blood urea nitrogen (BUN), calcium, chlorine, potassium, magnesium, sodium and phosphorus were measured using an automated analyzer (Accu-check Performa, Roche, Germany).

### 4.7. Urine Parameters

The contents of urine protein (UP), urine creatinine (UCr), calcium, phosphorus and magnesium were tested by the inspection center of the First Affiliated Hospital of Anhui University of Traditional Chinese Medicine.

### 4.8. Statistical Analysis

Results are expressed as the mean ± SD. Differences between groups were assessed by one-way ANOVA. Statistical tests were performed using SPSS software. Difference was considered statistically significant at *p* < 0.05 and *p* < 0.01. * *p* < 0.05, ** *p* < 0.01 versus control group; # *p* < 0.05, ## *p* < 0.01 versus CRF group.

## 5. Conclusions

In summary, experimental evidence in the present work confirmed that LJP61A has the ability to improve the renal function of adenine-induced CRF mice. Based on the current investigation, it is probable that the purified *L. japonica polysaccharide* LJP61A might be developed as a new therapeutic agent or functional food supplement to delay CRF in the future.

## Figures and Tables

**Table 1:** LJP61A regulates the electrolyte disturbance of adenine-induced CRF mice.

Group	Serumk Calcium (mmol/L)	Serum Chlorine (mmol/L)	Serum Potassium (mmol/L)	Serum Magnesium (mmol/L)	Serum Sodium (mmol/L)	Serum Phosphorus (mmol/L)	Urine Calcium (mmol/L)	Urine Phosphorus (mmol/L)	Urine Magnesium (mmol/L)
Control	3.27 ± 0.12	85.29 ± 0.26	9.10 ± 0.21	1.88 ± 0.01	146.97 ± 0.39	3.52 ± 0.16	2.84 ± 0.68	14.37 ± 2.8	4.01 ± 0.52
CRF	2.55 ± 0.05 **	105.97 ± 0.52 **	11.83 ± 0.12 **	2.43 ± 0.07 **	162.07 ± 1.76 **	5.82 ± 0.11 **	1.69 ± 0.30 *	8.75 ± 1.6 *	2.67 ± 1.78 *
Positive	3.13 ± 0.10 ^▲▲^	101.69 ± 0.61	9.60 ± 0.47 ^▲▲^	2.18 ± 0.04 ^▲▲^	148.15 ± 0.85	3.93 ± 0.25 ^▲▲^	2.56 ± 0.22 ^▲▲^	10.30 ± 3.5 ^▲▲^	3.10 ± 1.25 ^▲▲^
CRF+LJP61A50	2.56 ± 0.11	105.30 ± 0.27	10.43 ± 0.42 ^▲▲^	2.11 ± 0.12 ^▲▲^	158.73 ± 1.66	5.22 ± 0.12 ^▲▲^	1.91 ± 0.12 ^▲^	10.26 ± 2.1 ^▲▲^	2.89 ± 2.01 ^▲▲^
CRF+LJP61A100	2.78 ± 0.11 ^▲▲^	104.13 ± 0.66	10.07 ± 0.50 ^▲▲^	1.97 ± 0.07 ^▲▲^	152.66 ± 0.22	4.79 ± 0.13 ^▲▲^	1.89 ± 0.45 ^▲^	11.35 ± 1.9 ^▲▲^	2.92 ± 1.34 ^▲▲^
CRF+LJP61A200	2.98 ± 0.12 ^▲▲^	102.00 ± 0.90	9.97 ± 0.22 ^▲▲^	1.89 ± 0.06 ^▲▲^	150.73 ± 0.55	3.72 ± 0.22 ^▲▲^	2.49 ± 0.27 ^▲▲^	11.84 ± 3.0 ^▲▲^	3.05 ± 2.70 ^▲▲^

* *p* < 0.05, ** *p* < 0.01 (vs. Control group); ^▲^ *p* < 0.05, ^▲▲^ *p* < 0.01 (vs. CRF group).
